# 肺楔形切除术及纵隔淋巴结采样治疗早期高龄肺癌患者的体会

**DOI:** 10.3779/j.issn.1009-3419.2012.01.10

**Published:** 2012-01-20

**Authors:** 伟华 楼, 晓洪 詹, 信青 项, 伟 管

**Affiliations:** 322000 义乌，温州医学院附属义乌医院胸外科 Department of Thoracic Surgery, Yiwu Hospitol of Wenzhou Medical College, Yiwu 322000, China

**Keywords:** 肺肿瘤, 胸腔镜, 楔形切除, 淋巴结采样, Lung neoplasms, Video-assisted thoracoscopic surgery, Wedge resection, Mediastinal lymph node sampling

## Abstract

**背景与目的:**

治疗早期非小细胞肺癌首选手术治疗，但最佳的淋巴结清扫范围仍然不清楚。本研究旨在探讨肺楔形切除+纵隔淋巴结采样治疗在早期高龄肺癌患者中的价值。

**方法:**

2004年6月-2008年2月，共有15例70岁以上周围型肺癌患者接受胸腔镜下肺楔形切除术+纵隔淋巴结采样治疗。右侧肺癌行2R、4R、8及9组淋巴结采样，左侧肺癌行5、6、8及9组淋巴结采样。

**结果:**

早期高龄肺癌患者行胸腔镜下肺楔形切除术+淋巴结采样，手术时间短、创伤小，术后并发症少，恢复快。术后1年生存率和3年生存率分别为100%和86.6%。

**结论:**

肺楔形切除术+纵隔淋巴结采样治疗早期高龄周围型肺癌患者是一种选择。

## Abstract (en)

**Background and objective:**

The treatment for early-stage non-small cell lung cancer is primarily surgical. However, the optimal extent of mediastinal lymph node resection remains to be determined. The aim of the current study is to investigate the effect of a pulmonary wedge resection on aged patients with lung cancer.

**Methods:**

A total of 15 lung cancer patients aged over 70 years were treated via video-assisted thoracoscopic surgery (VATS) wedge resection and mediastinal lymph node sampling from June 2004 to February 2008. Mediastinal lymph node sampling included stations 2R, 4R, 8 and 9 for the right-sided cancers and stations 5, 6, 8 and 9 for the left-sided cancers.

**Results:**

VATS wedge resection and mediastinal lymph node sampling for aged patients with peripheral lung cancer are minimally invasive treatments with a short operative time and low postoperative morbidity. The 1-year and 3-year survival rates were 100% and 86.6%, respectively.

**Conclusion:**

VATS wedge resection and mediastinal lymph node sampling can be an alternative treatment for aged patients with early peripheral lung cancer.

在我国肺癌发病率居恶性肿瘤第三位，是最常见的肿瘤致死原因之一。老年人肺癌患者常并发各种慢性疾病，生理机能全面退化。在早期肺癌患者的外科治疗中，有限的肺切除是否符合肺癌的治疗原则仍有争论。温州医学院附属义乌医院胸外科自2004年6月-2008年2月共有15例70岁以上周围型早期肺癌患者接受胸腔镜下肺楔形切除+纵隔淋巴结采样治疗，全组患者术后并发症少，恢复较快，淋巴结采样结果阳性率低，1年、3年生存率与文献报道的肺叶切除+系统性淋巴结清扫结果接近。现就该组临床资料治疗结果及有关问题报告如下。

## 1 资料与方法

### 1.1 一般资料

本组病例15例，男12例，女3例；年龄75岁-88岁，平均年龄80.6岁。术前胸部增强CT考虑周围型占位，瘤体最大径＜2 cm，无明显肺门、纵隔淋巴结肿大；其中右上肺5例，右中肺叶1例，右下肺2例，左上肺3例，左下肺4例。入组标准：FEV1＞1.6 L，半年内无心肌梗死病史，无严重心律失常，无心功能不全，无远处转移，患侧无明显胸膜粘连，无胸膜炎病史。

### 1.2 手术方法

全组均采用全麻双腔管气管插管，健侧卧位，单肺通气。患侧腋中线第7肋间为观察孔，腋前线第3或4肋间及肩胛下角线第9肋间作两处切口为操作孔，在卵圆钳帮助下探查肿块位置，探查方法是一看二拨三指探。一看是指肿块表面有无粘连及胸膜凹陷；二拨是指用卵圆钳在肺表面轻轻滑拨，如遇到肿块时有异常；三指探是指上述方法仍无法判断肿块位置时，可以在卵圆钳的帮助下，伸入手指触摸探查以确认肿块位置。术中尽量避免钳夹肿块，以免肿瘤细胞入血。若肿块＜1 cm，术前在CT引导下行肿块定位。术中应用胸腔镜直线型切割吻合器（Endo-GIA，美国强生公司）距恶性肿瘤1 cm以上切割吻合肺组织，行肺楔形切除术，标本自切口取出（使用标本袋取出）。术中均行淋巴结采样，即在各组相应部位打开纵隔胸膜探查，若可见明确的淋巴结，则将淋巴结完整切除，若未见有明确淋巴结，则仅采取部分脂肪组织送检，而不作系统性淋巴结清扫。每组分别标记并送病理检查。右侧肺癌采样2R、4R、8及9组淋巴结，左侧肺癌采样5、6、8及9组淋巴结。因第7组淋巴结及4L组淋巴结位置较深，胸腔镜下暴露较困难而未采样。手术时间为65 min-122 min。

## 2 结果

15例肿瘤均完整切除、无破裂，完整取出标本的边界距肿瘤边缘＞1 cm，术后病理记录切缘未见癌细胞侵犯，无中转开胸；术后病理分型：鳞癌10例，腺癌5例。全组无围手术期死亡，术后无肺泡胸膜瘘等严重并发症发生。术后淋巴结采样病理证实N2淋巴结阳性1例，占6.7%。N2淋巴结阳性者，术后行辅助化疗，其余患者术后均未行放化疗等后续治疗。

术后随访至2011年2月，目前仍有11例存活。术后1年、3年生存率分别为100%、86.6%。由于病例数较少，并且有些病例术后随访时间不足5年，5年生存率还有待继续随访。

## 3 讨论

肺癌治疗的金标准为肺叶切除术+系统性纵隔淋巴结清扫。系统性淋巴结清扫要求按照淋巴结分组逐一清除各组淋巴结及周围脂肪组织，每组分别标记并送病理检查。右侧手术淋巴结清扫范围应包括1组-4组、7组-12组，左侧至少包括2组-12组淋巴结。金标准手术创伤大，术后引流相对较多，不适用于老年患者，尤其是有心肺功能不全的老年患者。

局部切除治疗早期肺癌的观点仍在探讨中。关于局部切除治疗早期肺癌最值得我们关注的有以下几个问题：早期肺癌局部切除疗效如何？是否需要行淋巴结清扫？是否需要淋巴结完全切除？淋巴结清扫对远期疗效的意义有多大？局部切除后的综合治疗，包括化学治疗及放射治疗等。不断有学者在这些方面进行探索。Okami等^[1]^报道早期高龄肺癌患者行肺局部切除能够取得与肺叶切除相同的生存率。研究发现75岁以上早期高龄肺癌患者行肺叶切除与肺局部切除，5年生存率无明显差异，而年轻组则有明显差异。Sugi等^[2]^报道了胸腔镜下局部切除治疗早期肺癌的远期疗效，A组为瘤体大小（T）＜1.5 cm行肺楔形切除，B组为T介于1.5 cm-2 cm，行肺段切除+淋巴结采样，C组为T介于2.0 cm-3.0 cm，行肺叶切除并淋巴结清扫。术后三组的无复发5年生存率分别为100%、90.5%和94.5%。Kates等^[3]^认为早期≤1 cm的非小细胞肺癌行局部切除可以取得与肺叶切除相同的疗效。因为术后并发症少并且术后可较好地保留肺功能，选择局部切除治疗小肿瘤也许更为合适。研究还发现肿瘤大小介于2 cm-3 cm的，宜选择系统性淋巴结清扫，若肿瘤＜2 cm，可以选择淋巴采样以减少创伤。Ishiguro等^[4]^发现选择性淋巴结清除与完全性淋巴结清扫组的5年生存率分别为76.0%和71.9%，选择性淋巴结清除并未使存活率下降。Darling等^[5]^选择无肺门淋巴结转移的T1或T2非小细胞肺癌病例，行淋巴结采样（右侧2R、4R、7、10R，左侧5、6、7、10L）与淋巴结清扫两组对照研究，结果淋巴结采样组5年生存率为69%，淋巴结清扫组为68%，两组无明显差异。

对70岁以上肺癌手术治疗选择，临床上应根据患者心肺功能、肿瘤部位、大小、病理类型及支气管受侵情况来确定手术方式。70岁以上老年患者单纯肺楔形切除或肺段切除是否能取得或接近上述金标准的结果。国内外一些学者应用局限性肺切除治疗心肺功能低下且不能耐受切除手术的周围型早期肺癌，结果令人鼓舞。Rami-Porta等^[6]^报道＞71岁的肺癌患者行肺楔形切除与肺叶切除的存活率相近。对于肿瘤＜2 cm的患者，肺楔形切除或肺段切除时切缘距离肿瘤＞1 cm的，可以避免或减少术后局部复发。Mun等^[7]^报道了胸腔镜手术治疗80岁以上高龄临床Ⅰ期肺癌的疗效，55例80岁以上高龄肺癌患者通过胸腔镜下行肺叶、肺段及肺楔形切除术，术后3年生存率为76.4%，5年生存率为64.9%，其中10例患者因FEV1＜0.81，故选择单纯肺楔形切除，报道指出由于胸腔镜技术的不断成熟，80岁以上高龄患者行胸腔镜下淋巴结切除术的风险较低，且认为胸腔镜下淋巴结清扫不增加术后并发症，对术后长期生存有利。Wisnivesky等^[8]^报道了＞65岁的Ia期T≤2肺癌患者行局部切除（肺段或肺楔形切除）或叶切除术疗效相近，认为有限的切除术也许是这些患者有效的治疗选择。Takizawa等^[9]^报道临床Ⅰ期非小细胞肺癌行系统淋巴结清扫组术后5年生存率为78.0%，淋巴结采样组术后5年生存率为76.2%，临床Ⅰ期非小细胞肺癌淋巴结采样与系统淋巴结清扫疗效相当。Kodama等^[10]^认为临床诊断为直径＜2 cm的早期肺癌不需行系统性纵隔或肺门淋巴结清扫。Okada等^[11]^通过多中心大型试验得出结论：临床Ⅰ期非小细胞肺癌行选择性纵隔淋巴结清除术可以达到与系统性纵隔淋巴结清扫一样的结果，并可作为手术微创外科治疗的一种选择。Williams等^[12]^通过对过去6年期间T1N0M0患者回顾性分析得出：Ⅰ期肺癌患者2年生存率为91%，5年生存率为80%，研究还发现Ⅰ期肺癌患者生存率很高，除TNM分期外，手术年龄、性别及手术范围对术后的存活率至关重要。

正因为国内外报道意见不一致，目前对于如何为老年高龄早期周围型肺癌患者选择最为恰当的手术方式尚无定论。我们对70岁以上高龄早期周围型Ⅰ期肺癌患者选择楔形切除+纵隔淋巴结采样的术式进行探索。本组15例患者均采用胸腔镜下肺楔形切除术+淋巴结采样，术中出血少，手术时间及麻醉时间短，术后拔管时间短，早期能下床活动，术后并发症少，恢复顺利，全组均未出现围手术期死亡病例。术后随访结果：术后1年、3年生存率分别为100%和86.6%，较文献报道高。5年生存率因为随访时间相对较短，有待进一步随访。我们认为针对早期高龄肺癌患者，尤其是合并有心肺功能不全的老年患者，选择胸腔镜下肺楔形切除+纵隔淋巴结采样取得了良好的治疗效果，是一种微创的手术。
